# Differing Expression and Potential Immunological Role of C-Type Lectin Receptors of Two Different Chicken Breeds against Low Pathogenic H9N2 Avian Influenza Virus

**DOI:** 10.3390/pathogens13010095

**Published:** 2024-01-22

**Authors:** Sungsu Youk, Dong-Hun Lee, Chang-Seon Song

**Affiliations:** 1 Microbiology Laboratory, Department of Medicine, College of Medicine, Chungbuk National University, Cheongju 28644, Republic of Korea; sungsu.youk@chungbuk.ac.kr; 2 Wildlife Health Laboratory, College of Veterinary Medicine, Konkuk University, Seoul 05029, Republic of Korea; donghunlee@konkuk.ac.kr; 3 Avian Diseases Laboratory, College of Veterinary Medicine, Konkuk University, Seoul 05029, Republic of Korea

**Keywords:** C-type lectin receptor, chicken, Lohmann Brown, Lohmann White, avian influenza, H9N2

## Abstract

Diverse immune responses in different chicken lines can result in varying clinical consequences following avian influenza virus (AIV) infection. We compared two widely used layer breeds, Lohmann Brown (LB) and Lohmann White (LW), to examine virus replication and immune responses against H9N2 AIV infection. The transcription profile in the spleen of H9N2-infected chickens was compared using a microarray. Confirmatory real-time RT-PCR was used to measure the expression of *C-type lectin*, *OASL*, and *MX1* genes. Additionally, to investigate the role of chicken lectin receptors in vitro, two C-type lectin receptors (CLRs) were expressed in DF-1 cells, and the early growth of the H9N2 virus was evaluated. The LB chickens shed a lower amount of virus from the cloaca compared with the LW chickens. Different expression levels of *C-type lectin-like* genes were observed in the transcription profile, with no significant differences in *OASL* or *MX* gene expression. Real-time RT-PCR indicated a sharp decrease in C-type lectin levels in the spleen of H9N2-infected LW chickens. In vitro studies demonstrated that cells overexpressing CLR exhibited lower virus replication, while silencing of homeostatic CLR had no effect on AIV replication. This study demonstrated distinct immune responses to H9N2 avian influenza in LB and LW chickens, particularly with differences in C-type lectin expression, potentially leading to lower virus shedding in LB chickens.

## 1. Introduction

Avian influenza (AI) is a devastating disease that can cause serious economic damage to the poultry industry. H9N2 subtype low pathogenic avian influenza (LPAI) viruses are endemic in many countries throughout Asia, the Middle East, and Northern Africa [1]. Although H9N2 viruses are not highly pathogenic, high morbidity and moderate mortality have been observed when hosts are co-infected by bacterial pathogens, other viral pathogens, or even live vaccine viruses [2,3,4]. In addition, H9N2 viruses have been recognized as major donors providing internal genes in the evolution of H5 highly pathogenic avian influenza viruses. Thus, better control of H9N2 viruses is required to prevent the emergence of new zoonotic viruses [5].

Early innate immunity determines disease outcome and the pathogenesis of AI infection as the first line of the immune response. As domestic chickens are bred to inherit better productivity, selection for high production efficiency is often associated with undesirable side effects, such as deficiencies in physiological, immunological, or reproduction traits and immunological competence to infectious diseases [6,7]. We have demonstrated that immunocompromised chickens infected with H9N2 show elevated levels of viral RNA in peripheral blood lymphocytes with viral proteins in the kidney, particularly in chickens that succumbed to viral infection [8]. Later, in a study using specific pathogen-free chickens, increased transcription of interferon genes and related immune response genes such as 2′-5′-oligoadenylate synthetase and myxovirus resistance genes were found to be upregulated in response to H9N2 infection [9].

The C-type lectin superfamily consists of numerous proteins having one or more carbohydrate moieties in a calcium-dependent manner using conserved carbohydrate recognition domains. C-type lectin receptors (CLRs) on immune cells are involved in the recognition and induction of innate and adaptive immune as one of the pattern recognition receptors [10]. While C-type lectins secreted in the lungs have been studied for their roles in antiviral immunity against influenza virus by directly binding to viral surface protein or supporting adaptive immunity in complement activation in mice [11] and chickens [12], little is known about the role of chicken CLRs in antiviral immunity against avian influenza virus (AIV) infection.

The Korean poultry industry has suffered from a distinct lineage of the H9N2 LPAI virus, which is endemic in South Korea. Since their emergence, Korean lineage H9N2 viruses have evolved to adapt to domestic chickens through antigenic drift and shift with wild bird origin AI viruses [13,14]. Although many studies have focused on virus evolution and pathogenesis of H9N2, host factors in different chicken breeds in response to infection by Korean lineage H9N2 virus have not been compared yet. Thus, the objective of this study was to compare immune responses and viral shedding patterns in two common layer breeds in Korea when they were infected with a Korean lineage H9N2 LPAI virus. To further corroborate the antiviral effect of the most differentially expressed gene in vitro, chicken CLR was cloned and expressed in chicken embryo fibroblast cells to evaluate their viral resistance to H9N2 virus infection.

## 2. Materials and Methods

### 2.1. Animal Experiment

Chicken experiments were reviewed and approved by the Institutional Animal Care and Use Committee of Konkuk University (KU11034). Animal infection was conducted in an animal biosafety level 2 facility, as approved above. Three-week-old Lohmann Brown (LB) and Lohmann Leghorn White (LW) chickens were obtained from commercial chicken farms to compare H9N2 virus replication in different layer breeds. These chickens were tested serologically negative for type A influenza using nucleoprotein (NP)-ELISA (Bionote, Korea). Ten chickens from each breed were intrachoanally inoculated with 10^6^ egg infection dose 50 percent (EID_50_) H9N2 LPAI virus (A/Korean native chicken/Korea/K040110/2010, GenBank accession No. JF917100- JF917107) in 0.1 mL sterile phosphate-buffered saline (PBS) and three chickens were intrachoanally inoculated with 0.1 mL of PBS as a control for each breed. Inoculated chickens were separately housed by breed. Swabs from oropharyngeal (OP) and cloacal (CL) routes were collected at 3, 5, and 7-day-post infections (dpi) and examined for virus shedding. The selection of swabbing days was informed by a prior study, which observed the peak virus shedding titer at 5 dpi under the same experiment setting [15]. Post-infection sera were collected at 14 dpi, and seroconversion was examined using the NP-ELISA kit (Bionote).

The same infection schedule was set to measure gene expression levels related to antiviral immunity. Nine chickens of each breed were inoculated as executed for swab chickens, and nine chickens of each breed were inoculated with PBS as a control. Three chickens of each group were euthanized at 1, 3, and 5 dpi. Spleen collection days were determined based on our previous study, where innate antiviral response genes (*OASL* and *Mx1*) were increased at 1 dpi and then diminished completely by 5 dpi [9].

Their spleens were collected and stored at −80 °C until total RNA extraction. Total RNAs were extracted using a RNeasy Mini kit (Qiagen, Germany) according to the manufacturer’s protocol.

### 2.2. Virus Shedding

Viral RNA was extracted from 150 µL of OP and CL swab sample suspension using a Viral Gene-Spin viral DNA/RNA extraction kit (iNtRon Biotechnology, Korea). Real-time reverse-transcription polymerase chain reaction (rRT-PCR) targeting the Matrix gene was performed as previously described [16]. For extrapolation of cycle threshold (Ct) values to infectious units, serial dilutions of the inoculum were calculated as EID_50_/mL. Corresponding virus doses were analyzed by rRT-PCR. The resulting calibration curves were highly predictable (R^2^ = 0.99) and used for converting Ct values to EID_50_/mL.

### 2.3. Preparation of Fluorescent RNA Probe, Hybridization, and Analysis

The total RNA extracts from the spleen samples were used for microarray analysis. Each total RNA sample (200 ng) was labeled and amplified using a Low Input Quick Amp labeling kit (Agilent Technologies, Santa Clara, CA, USA). Cy3-labeled aRNAs were resuspended in 50 μL of hybridization solution (Agilent Technologies). After labeled aRNAs were placed on Agilent SurePrint G3 chicken GE 8 × 60 K array (Agilent Technologies) and covered by a Gasket 8-plex slide (Agilent Technologies), slides were hybridized at 65 °C in an oven for 17 h. These hybridized slides were washed with 2 × SSC, 0.1% SDS for 2 min, 1× SSC for 3 min, and 0.2× SSC for 2 min at room temperature. Slides were then centrifuged at 3000 rpm for 20 s to dry.

Arrays were analyzed using an Agilent scanner with associated software. Gene expression levels were calculated with Feature Extraction v10.7.3.1 (Agilent Technologies). Relative signal intensities for different genes were generated using the Robust Multi-Array Average algorithm. Data were processed based on the median polish normalization method using GeneSpring GX 11.0 (Agilent Technologies). This normalization method aims to make the distribution of intensities for each array in a set of arrays the same. Normalized and log-transformed intensity values were then analyzed using a GeneSpring GX 11.0 (Agilent Technologies). Fold change filters for the genes were set to be present in at least 200% of controls for upregulated genes and lower than 50% of controls for down-regulated genes. Hierarchical clustering was performed for groups that behaved similarly across experiments using GeneSpring GX 7.3.1 (Agilent Technologies). The clustering algorithm was Euclidean distance and average linkage.

### 2.4. Quantification of Chicken C-Type Lectin, OASL, and MX1 mRNA

Three innate immune-related genes in splenic total RNA were quantified by real-time reverse transcriptase polymerase chain reaction (rRT-PCR) in a thermal cycler (Smart Cycler System, Cepheid, Sunnyvale, CA, USA) using a One-Step SYBR Green Master Mix II (Takara, Dalian, Jiangsu, China) according to the manufacturer’s instructions. Primer sets for quantification of chicken C-type lectin (chC-lectin), chicken 2′-5′-oligoadenylate synthetase-like (chOASL), chicken myxovirus resistant 1 (chMx), and chicken GAPDH (chGAPDH) mRNAs are listed in Table S1. The transcription level of mRNA was normalized using the comparative 2^−ΔΔCt^ method, which was used to determine the mean fold increase in the expression level of the respective genes from corresponding uninfected chickens [17].

### 2.5. Cloning and Expression of Chicken C-Type Lectin

Based on the rRT-PCR result that the C-type lectin-related gene was differentially expressed between LB and LW, primer sets were designed to amplify the corresponding mRNA of CLR (GenBank accession NM_001393724.2) using reverse transcriptase polymerase chain reaction (Table S2). cDNA synthesis for total RNA prepared from peripheral blood mononuclear cells from a LB chicken provided from the same commercial farm that provided the LB chickens for the infection study. Plasmid construction was performed using a previously established method [18]. Briefly, the coding sequences of two CLRs (CLR1 and CLR2) were inserted into the pcDNA3.1/His A vector (Invitrogen, Darmstadt, Germany), resulting in the addition of a polyhistidine tag at the N-terminal end when translated. To determine CLR expression, DF-1 (chicken fibroblast, ATCC CRL-12203) was transfected with 0.5 μg of CLR-encoding pcDNA3.1/His A vector (pcDNA-CLR1 or pcDNA-CLR2) and empty plasmid (pcDNA3.1) using Lipofectamine 2000 (Invitrogen). The cells and medium were harvested at 12 h post-transfection. Cell lysate and supernatant were analyzed by Western blotting using mouse anti-6X His tag antibody (Abcam, Cambridge, UK) and peroxidase-conjugated goat anti-mouse monoclonal secondary antibody (Santa Cruz Biotechnology, Santa Cruz, CA, USA). To locate the expression of CLRs, transfected DF-1 cells were subjected to immuno-fluorescence assay using mouse anti-6X His tag antibody (Abcam) and Alex Fluor 488 anti-mouse secondary antibody (Invitrogen).

### 2.6. Virus Infection and Titration In Vitro

H9N2 virus was titrated in Madin-Darby canine kidney (MDCK) cells (ATCC, CRL-34). CLR1- or CLR2-expressing DF-1 cells were inoculated with the H9N2 virus at a multiplicity of infection of 0.025. After 12 h post-inoculation, culture supernatants were harvested, and viral load in each supernatant was measured as tissue cell infection dose 50 (TCID_50_) in MDCK cells.

### 2.7. Silencing of Chicken C-Type Lectin

Two small interfering RNA (siRNA) targeting CLRs [CLR-siRNA-113: 5′- GAGCUGCACACAGGAUCUCC (dTdT)-3′, and CLR-siRNA-216: 5′- CAAAGGCUGAGAAAGGUGGG (dTdT)-3′] and a validated negative control siRNA [Negative control: 5′-CCUACGCCAAUUUCGU-3′] were designed and synthesized by ST Pharm Co., Ltd. Small interfering RNA (siRNA) transfection was conducted using Lipofectamine 2000 (Invitrogen) at a final concentration of 50 mM. Knockdown of CLR mRNA was confirmed by the same rRT-PCR method used for chC-lectin quantification. At 12 h post-transfection, H9N2 viruses were inoculated at an MOI of 0.025. Cells were harvested at 12 h post-infection, and viral load in the supernatant was measured as TCID_50_ in MDCK cells.

### 2.8. Statistical Analysis

The difference in the number of CL viruses shedding between LB and LW was tested by Fisher’s exact test in Excel. Differentially expressed genes of CLR between LB and LW and two upregulated antiviral genes of *chOASL* and *chMx* were tested using a *t*-test implemented in GraphPad Prism 9.0. Comparison of viral growth in vitro was conducted using a one-way analysis of variance and Tukey’s post hoc comparison in Origin 2022 software (ver. 9.9.0.225).

## 3. Results

### 3.1. Virus Shedding in LB and LW Chickens following Infection with Korean H9N2 LPAI Virus

**Figure 1:**
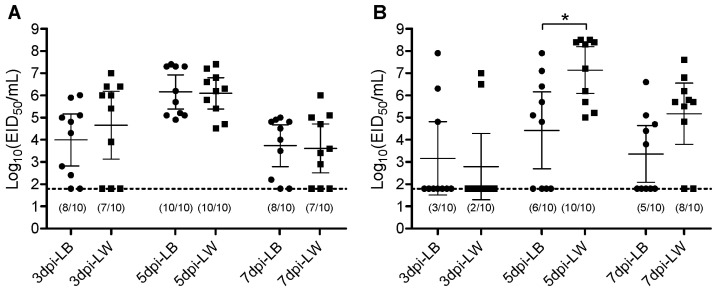
Scatter plot of oropharyngeal (**A**) and cloacal (**B**) virus shedding detected by rRT-PCR from LB and LW chickens inoculated with H9N2 LPAI virus. Virus titers are expressed as log10 with error bars. For statistical purposes, rRT-PCR-negative samples were given a value of the limit of detection (1.8 log_10_ EID_50_/mL). The circle (●) and square (■) plots indicate viral shedding from LB and LW chickens, respectively. The asterisk indicates the significance of the mean virus titer of 5 dpi oropharyngeal swab between LB and LW (*p* < 0.001). The numbers of rRT-PCR-positive samples are indicated in parenthesis (virus shedding positive/total).

The Korean lineage H9N2 virus was intrachoanally inoculated to each of the ten LW or LB layers. Oropharyngeal (OP) and cloacal (CL) virus swabs were taken at 3, 5, and 7 dpi. Viral genes were quantitively measured by rRT-PCR. Amounts of viral shedding were calculated by extrapolating to EID_50_ unit. All chickens of both breeds became infected based on OP virus shedding and serology results at 14 dpi. In the serological analysis conducted with NP ELISA, all chickens from both breeds tested seropositive. The serum antibody titers, represented by the percentage inhibition values, did not exhibit significant differences between the breeds. However, the number of chickens with CL virus shedding differed between breeds. All ten LW chickens shed the virus via the CL route, whereas only six out of ten LB chickens shed the virus (Table 1). No difference in mean virus shedding titer was observed in the OP route throughout the swab collection days. In the CL route, the LW chickens shed a greater amount of virus than the LB chickens at 5 dpi (Figure 1). This higher virus shedding in the LW group corresponds with the greater number of chickens shedding the virus. These results indicate that while infectivity of the H9N2 virus is similar to both LB and LW chickens, its replication and virus shedding are less effective in LB chickens than in LW chickens.

### 3.2. C-Type Lectin-Associated mRNA Genes Are Differentially Transcribed in Spleen of LW and LB Breeds

**Figure 2:**
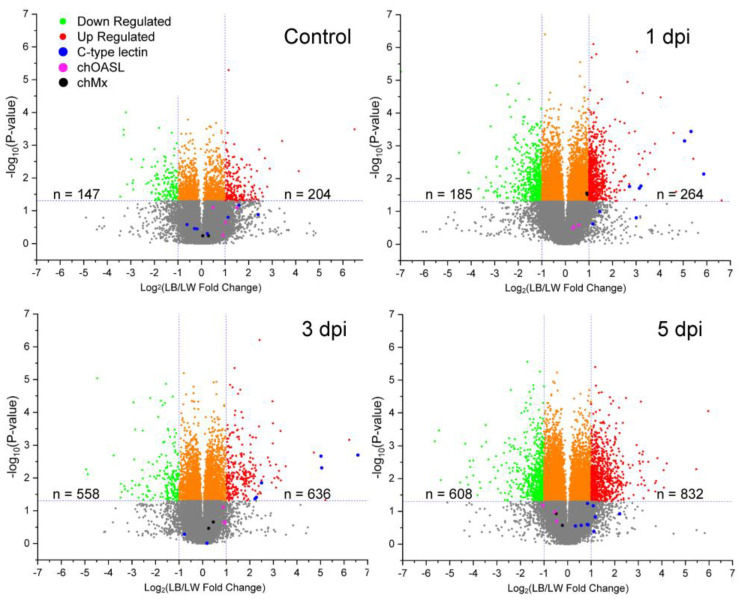
Relative transcriptional gene expression profile in spleen of H9N2 infected LB and LW by microarray. H9N2 virus was inoculated into LB and LW layers. Spleen samples were collected from three chickens of each breed at each time point (1, 3, and 5 dpi). Relative gene expression of LB was measured by calculating LB/LW fold change. Differentially expressed genes were considered when criteria for fold change and *p*-value (fold change greater than 2 or smaller than 0.5 with *p*-value less than 0.05) were met. The number of differentially expressed genes was counted. C-type lectin-like, chOASL, and chMx mRNA are colored in blue, fuchsia, and black, respectively.

Transcriptional profile was examined to identify genes differentially expressed between LW and LB chickens in response to H9N2 virus infection. Each breed of three uninfected control and three H9N2-infected birds was sacrificed to collect spleen tissue at 1, 3, and 5 dpi. Relative fold change of LB/LW gene transcription was visualized as a scatter plot (Figure 2). Differential gene expression was considered significant if LB/LW fold change was greater than 2 or smaller than 0.5 with a *p*-value less than 0.05. In contrast to the uninfected control group, a higher *C-type lectin-like* gene expression profile was observed in LB as compared with LW. Six of nine *C-type lectin-like* genes showed higher gene expression levels at 1 and 3 dpi. In a previous study, *chOASL* and *chMX* were the two antiviral genes that were most greatly increased in White Leghorn chickens upon H9N2 infection [9]. However, no significant difference in upregulation of chOASL and chMX mRNA was observed between the two breeds used in this study, although the chMx mRNA showed a marginally higher gene expression in LB at 1 dpi.

### 3.3. Expression Levels of Splenic C-Type Lectin Receptors Are Downregulated in LW Breed but Unchanged in LB Breed upon H9N2 Infection

**Figure 3:**
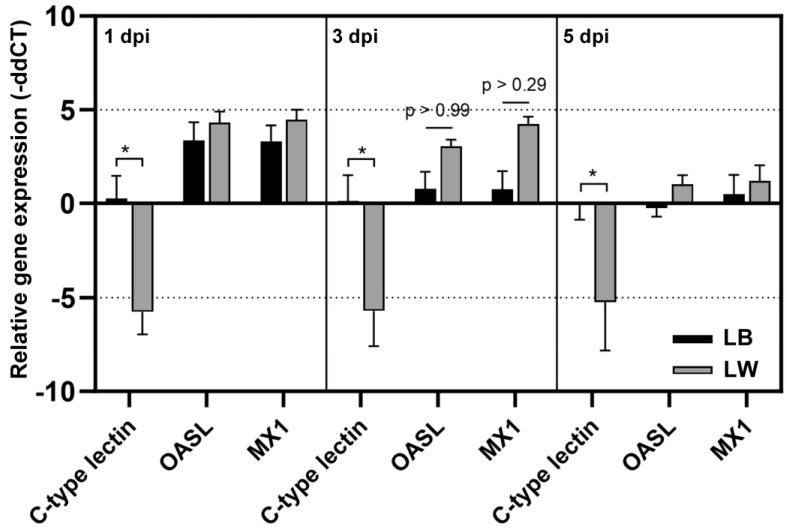
Differential mRNA level of chC-lectin, chOASL, and chMx. Quantitative real-time reverse transcriptase polymerase chain reaction was conducted to confirm relative mRNA expression revealed by microarray. The relative gene expression levels were quantified by calculating the mean fold change; gene expression levels at a given time point were compared to that of uninfected chickens. The asterisk indicates the significance of the gene expression between LB and LW (*p* < 0.001).

Three chickens of each group of LB and LW were sacrificed to collect spleen samples at 1, 3, and 5 dpi. chC-lectin, chOASL, and chMX mRNA levels in total RNA samples were measured by quantitative rRT-PCR (Figure 3). The chC-lectin level in LB remained similar to that in the control group throughout the sampling days. However, the chC-lectin level was downregulated approximately 32-fold in LW after H9N2 infection at all sampling days. In response to H9N2 infection, chOASL and chMX levels were increased in both breeds, showing no significant difference between the two breeds. An elevation in chOASL and chMX levels was noted in both H9N2-infected LW and LB chickens. However, a notable decrease in splenic chC-lectin levels was observed in LW chickens, in contrast to LB chickens, where these levels remained relatively unchanged.

### 3.4. Differentially Expressed C-Type Lectin Was C-Type Lectin Receptors Derived from Chicken Major Histocompatibility Complex Y Region

**Figure 4:**
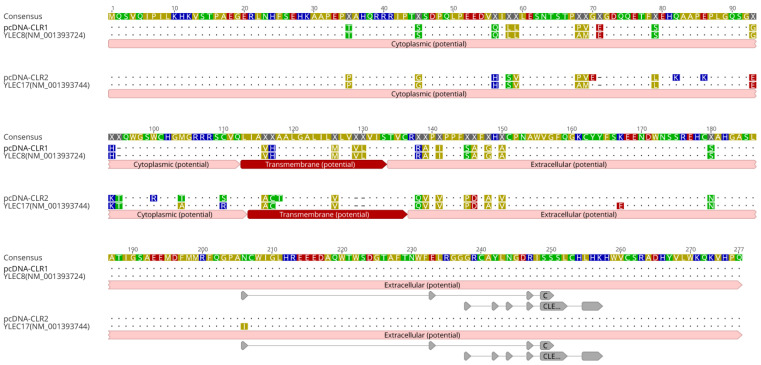
Amino acid sequences of cloned CLR1 and CLR2. C-type lectin receptor sequences were cloned into expression vectors. Amino acid sequences of cloned pcDNA-CLR1 and pcDNA-CLR2 were aligned and compared with two references, *YLEC8* (NM_0013937243.2) and *YLEC17* (NM_001393744). The letters in sequences are highlighted based on polarity of amino acids. Potential cytoplasmic, transmembrane, and extracellular regions were annotated with a prediction tool implemented in Geneious Prime v. 2023.0.4. Highlighted areas in pcDNA-CLR2 and *YLEC17* are different sequences with amino acid abbreviations. Dots indicate consensus sequences. Dashed areas are blank. For extracellular carbohydrate-binding domains, two conserved C-type lectin domains across eukaryotic cells were predicted using position-specific scoring matrix (PSSM ID: 153,057 and 153063). Gray arrows indicate predicted putative ligand binding sites.

Differentially expressed C-type lectin based in microarray and rRT-PCR corresponded to a wide variety of mRNA sequences of chickens CLR genes derived from major histocompatibility complex (MHC)-Y region in microchromosome 16. The MHC-Y region is genetically distant from the MHC-B region, encoding non-classical MHC class I and II genes [19,20]. A primer set targeting the coding region of chicken CLRs was designed based on coding sequences of *YLEC8* (accession No. NM_001393724) and *YLEC17* (NM_001393744) genes. To clone *CLR* genes from peripheral blood mononuclear cells of LB, cDNA synthesis and cloning to expression vector were conducted. The CLR sequences cloned to pcDNA3.1 were confirmed by comparing them to references (Figure 4). Sequences of pcDNA-CLR1 and pcDNA-CLR2 shared 99.28% and 95.99% identities with *YLEC8* and *YLEC17*, respectively. Putative ligand binding sites were localized as protein features annotated in the reference and a closely related C-type lectin-like protein isoform sequence (XP_046757382). Binding domains were similar to C-type lectin-like domains found in natural killer cell receptors (PSSM-Id: 153063) and unclassified C-type lectin-like domains (PSSM-Id: 153057) according to conserved domain hierarchical classifications [21]. Coding regions of CLR1 and CLR2 sequences were deposited under accession numbers of OR232705 and OR232706, respectively, in GenBank.

### 3.5. pcDNA-CLR1 and pcDNA-CLR2 Are Transiently Expressed in DF-1 Cells

**Figure 5:**
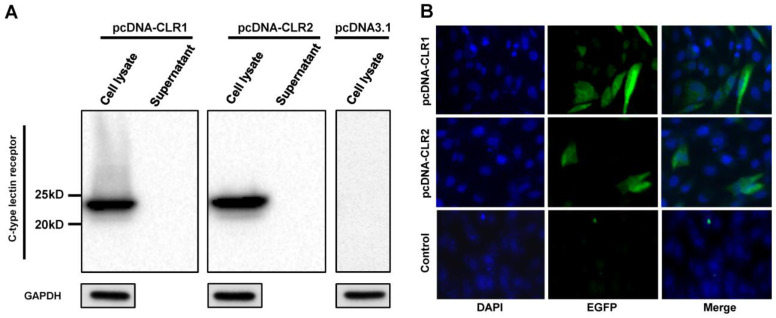
Expression of CLRs in DF-1 cells. Expression of CLRs were analyzed by Western blotting (**A**) and immunofluorescence assay (**B**). DF-1 cells were transfected with pcDNA-CLR1 or pcDNA-CLR2. Cell lysate and medium were then harvested at 12 h after transfection and analyzed by Western blot assay. Separately, localization of CLRs was demonstrated using mouse anti-6X His tag antibody and Alex Fluor 488 anti-mouse secondary antibody (green). Cell nuclei were stained with 4,6-diamidino-2-phenylindole (blue).

Expression of cloned CLR1 and CLR2 in DF-1 cells was verified in cell lysate and supernatant by Western blot (Figure 5A). Both CLRs were detected in cell lysate samples at 12 h after DF-1 transfection but not in supernatant samples, indicating that these CLRs were not secreted to cell media. The major band was detected at the 25 kDa band. The expression of CLRs was also verified by immunofluorescence assay (Figure 5B). The percentage of fluorescent cells was calculated from the image. The transfection efficiency was about 16–20% for pcDNA-CLR1 and 7–12% for pcDNA-CLR2. These results suggested that the cloned CLRs were expressed in transfected DF-1 cells, with a different efficiency while both did not release detectable levels of CLRs to the culture medium.

### 3.6. Early H9N2 Virus Growth Is Decreased in CLR-1-Expressing DF-1 Cells

**Figure 6:**
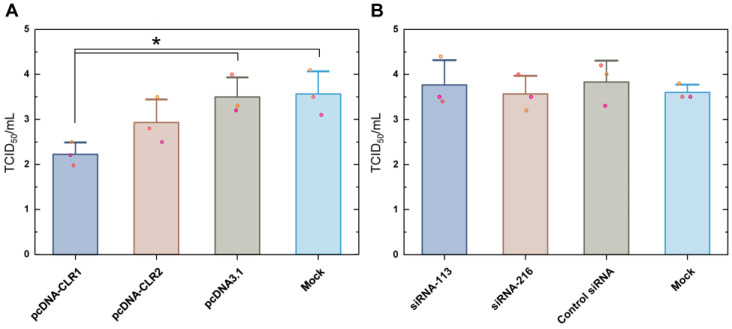
H9N2 virus growth under overexpression or downregulation of CLR in DF-1 cells. Transfection of CLR expressing plasmids (**A**) or siRNA targeting CLR mRNA (**B**) was conducted 12 h before inoculating virus. Supernatant virus titers were measured at 12 h after transfection using MDCK cells in triplicate. The asterisk indicates statistical significance by one-way ANOVA and Tukey’s post hoc comparison (*p* < 0.05).

We evaluated H9N2 growth in CLR1- or CLR2-expressing DF-1 cells as compared to the control vector-transfected or non-transfected DF-1 cells (Figure 6A). DF-1 cells were infected with H9N2 virus at 12 h after transfection. Supernatant samples were titrated in MDCK cells at 12 h post-infection. CLR1-expressing DF-1 cells showed a reduced viral titer of about 1.3 TCID_50_/mL as compared with both mock vector (pcDNA3.1) and transfection agent control (Mock) groups. Transfection of CLR2 marginally decreased mean virus titer. We also examined how a decreased level of constitutively expressed CLR level influenced H9N2 virus replication (Figure 6B). Two siRNAs were designed for silencing of CLR mRNA. Cells were transfected 12 h before H9N2 inoculation. Downregulation of baseline CLR before H9N2 infection did not change virus titer in any siRNA group. In summary, over-expression of CLR decreased early H9N2 replication in DF-1 cells, while downregulation of constitutively-expressing CLR did not affect virus growth.

## 4. Discussion

Diverse innate immune responses in various chicken lines can lead to markedly different clinical consequences against infectious diseases. The most pronounced disparities in response have been observed when examining the effects of salmonellosis, an infection caused by a bacterial pathogen, on different chicken lines. Natural resistance to specific serotypes of *Salmonella* spp. was observed in white chicken breeds, indicating that certain chicken lines might possess a general mechanism of resistance [22,23]. For viral diseases, highly inbred chicken lines have been identified to contain several genes known to be involved in the immune response to Marek’s disease caused by a virus infection, which potentially could contribute to higher survival and less tumor incidence [24].

Virus shedding after H9N2 LPAI virus infection clearly indicated different virus replication between two genetically different chicken breeds. While all LB and LW layers succumbed to H9N2 virus infection and shed virus in the oropharynx, a smaller number of LB shed virus in the cloaca than LW with a lower amount of virus shedding at 5 dpi. In line with our observation, the brown layer showed better resistance against virulent or intermediate pathotypes of the AI virus, with a lower amount of oropharyngeal virus shedding and a higher survival rate than the white layer, regardless of performance type [25]. In addition, brown layers had a faster CD8-positive immune cell response after viral or allogenic stimulus than white layers. A similar trend was also observed when virus replication was compared between Hy-Line brown and white. The difference in oropharyngeal virus titer was marginal. However, viral titers in the lung and cecal tonsil were higher in Hy-Line white for different genotypes of H9N2 viruses [26]. These series of findings provide the basis for follow-up studies to further examine the underlying mechanism of AI resistance between brown and white layers.

Available evidence indicates an involvement of secreted and circulating lectins in the immune response against respiratory viral infection in chickens. Heightened levels of lectin have been observed in chicken serum during respiratory viral infections, including infectious bronchitis virus and infectious laryngotracheitis virus [27]. Moreover, lung C-type lectin in chickens can exert inhibitory effects on the hemagglutination activity of human influenza A virus subtypes H3N2 and H1N1 [12]. Of significant interest, infection of chickens with H9N2 has been revealed to have a distinctive impact on lectin expression at the mRNA level, with variations influenced by age and location [28]. This finding highlights the interplay between viral infections and lectin-mediated immune responses, underscoring the potential of lectins as modulators in avian antiviral defense systems.

The role of chicken CLRs in AI virus infection remains largely unexplored. In this study, chicken CLRs were found to be differentially expressed between LB and LW in response to H9N2 infection. Unexpectedly, CLR expression was sharply decreased in LW while it remained consistent in LB. Chicken CLR genes are designated *Ylec* genes, similar to mammalian genes encoding C-type lectin-like receptors that could guide mammalian natural killer cell response [29]. However, whether it is more abundant in chicken natural killer cells as a C-type lectin-like receptor encoded by *Blec* genes from the MHC-B region [30] needs to be investigated in the future. *Ylec* genes belong to the MHC-Y region in microchromosome 16, which also encodes polymorphic non-classical class I genes along with non-classical class II B [20]. It is well-known that the MHC-B region is associated with viral disease susceptibility. MHC-B haplotype characterizes genetic resistance to oncogenic viral diseases, including Rous sarcomas and Marek’s disease [31,32,33]. However, regarding the MHC-Y haplotype, although birds homozygous for one MHC-Y haplotype were at 2.3 times greater risk for developing virus-induced tumors compared with all other MHC-Y genotypes combined [34], no major influence was found for MHC-B. Regarding other infectious viral diseases, different MHC-B haplotypes are associated with differing disease outcomes of highly pathogenic AI, chicken infectious laryngotracheitis, infectious bursal disease, and chicken infectious bronchitis [35]. However, association with MHC-Y is rarely understood. In particular, the role of *Ylec* is unknown.

Primer set used for confirmational rRT-PCR was designed to amplify all *YLEC* mRNAs that could be found in a public database, including YLEC9 (NP_001384395), *YLEC10* (NP_001380655), *YLEC12* (NP_001380656), *YLEC13* (NP_001382994), *YLEC18* (NP_001380675), *YLEC19* (QSE03662), *YLEC21* (QSE03743), *YLEC23* (QSE03748), *YLEC28* (QSE03758), *YLEC29* (QSE03759), *YLEC34* (NP_001380687), and many other C-type lectin-like NK cell receptor isoforms. Thus, we were able to compare overall *YLEC* expression but not within *YLEC*. The cloned CLR1 and CLR2, genetically close to *YLEC8* and *YLEC17*, might not reflect the antiviral effect of all *YLEC*. However, it was evident that overexpression of CLR1 reduced early H9N2 virus replication in CEF cells. The compatibility of CLR2′s expression and biological function in DF-1 cells may be limited, as the CLR cloned in this study were sourced from the LB, which originates from Rhode Island and White Rock breed lines. On the other hand, the DF-1 cell line is derived from the White Leghorn breed. This difference in genetic lineage between the source of CLR genes and the DF-1 cell line might affect the function of CLR2 in DF-1 cells.

## 5. Conclusions

In conclusion, our study demonstrated differing immune responses between LB and LW and their implications for H9N2 virus replication. Decreased expression levels of *C-type lectin-like* genes in LB in response to H9N2 suggests the potential role of CLR in reducing cloacal virus shedding, which was also supported by decreased virus replication in CLR-overexpressed DF-1 cell in vitro. Further research is warranted to unravel the underlying molecular pathways, identify key immunological factors, and explore key CLRs as targets for developing chicken breeds naturally resistant to AIVs. Such endeavors will contribute to a deeper understanding of the avian immune system and facilitate the development of effective strategies to control and prevent infectious diseases in poultry populations.

## Figures and Tables

**Table 1:** Virus shedding and serology of LB and LW layers infected with H9N2 LPAI virus.

Chicken Breed	Swab Route ^1^	Number of Virus Shedding Positive/Total (Mean Virus Titer) ^2^				Number of Seroconverted/Total
		3 dpi	5 dpi	7 dpi	Total	
LB	OP	8/10 (3.6)	10/10 (6.1)	8/10 (3.5)	10/10	10/10
	CL	3/10 (2.6)	6/10 (3.7)	5/10 (3.0)	6/10 *	
LW	OP	7/10 (4.1)	10/10 (6.0)	7/10 (3.3)	10/10	10/10
	CL	2/10 (2.3)	10/10 (7.0)	8/10 (4.7)	10/10	

^1^ OP, oropharyngeal; CL, cloacal; dpi, days post-infection; ^2^ Number of chickens with virus detection/number of chickens inoculated. Values in parentheses are geometric mean virus titer of chickens with virus detection (log EID_50_/mL). The limit of detection was set at 1.8 EID_50_/mL; * Statistical significance was calculated by Fisher’s exact test compared to number of positive LW chickens (*p* = 0.025).

## Data Availability

The coding region sequences of CLR1 and CLR2 that were used for overexpression in DF-1 were deposited in GenBank under accession numbers OR232705 and OR232706, respectively.

## Supplementary Materials

The following supporting information can be downloaded at: https://www.mdpi.com/article/10.3390/pathogens13010095/s1, Table S1: Primer sets used for quantification of chicken C-type lectin, OASL, and MX1 mRNA level; Table S2: Primer set used for C-type lectin receptors cloning to expression vector.
