# Supplementary material for: Differing Expression and Potential Immunological Role of C-Type Lectin Receptors of Two Different Chicken Breeds against Low Pathogenic H9N2 Avian Influenza Virus
Source: Pathogens. 2024 Jan 22;13(1):95. doi: 10.3390/pathogens13010095 (PMC10818356; doi:10.3390/pathogens13010095)
Supplement: Supplementary file 1 [file pathogens-13-00095-s001.zip › pathogens-2780218-supplementary.pdf]

Table S1. Primer sets used for quantification of chicken C-type lectin, OASL, and MX1 mRNA level.

**Target gene (GenBank Acc. No.)**	**Forward primer**	**Reverse primer**	**Size**
chC-lectin (CR391386.1)	TCACCAACTGGTTT-GAGCTG	GCAGACCCAGTGCTT-GTGTA	101
chMx1 (NM_204609)	GGAGCAAGTAAAC-GCCTGAG	TCTGCTGGTTAG-CAGCTTCA	163
chOASL (NM_205041)	GCGGTCTACGTGAA-GCTGTT	TTCGGCTTCAACATCTC CTT	158
chGAPDH (NM_204305)	CCCAG-CAACATCAAATGGG-CAGAT	TGATAACACGCTTAG-CACCACCCT	118

Table S2. Primer sets used for C-type lectin receptors cloning to pcDNA3.1 vectors.

Target gene (GenBank Acc. No.)	Forward primer	Reverse primer	Size
C-type lectin	CGGATCCGCCGCCAC-CATGCAGTCTGTG-CAAATACCAAT	AGAATTCTTGTGGGTG-CACCTTTTGC	841
